# On a New Test for Distinguishing the Russian, Indian, and English Rhubarbs

**Published:** 1853-04

**Authors:** John S. Cobb


*On a New Test for Distinguishing the Russian, Indian, and English Rhubarbs.* By Mr. John S. Cobb.—The absence of a good test for distinguishing the Russian from East Indian rhubarb has been too much felt and too often expatiated on to require any comment. The want of it has led to a great many analyses, undertaken with a view to the discovery of some constituent peculiar to one or the other species; I am not, however, aware of any successful results having, up to the present time, ensued from these researches, at least so far as concerns the discovery of any reagent capable of distinguishing the varieties of this drug.

Several tests have indeed been proposed, but none of these are to be relied upon. The grittiness, when chewed, caused by the presence of oxalate of lime in the Russian variety, is far from being a satisfactory test. I have met with specimens of Indian rhubarb possessing this quality to a great degree.

Geiger states that "iodized hydriodic acid" gives with a decoction of Russian rhubarb a green tint, with Indian a brownish color, and a deep red with the English rhubarb; but in my hands this test has proved singularly fallacious; with it I obtained a green color with the Russian, a dirty green with Indian, and a sea-green with English rhubarb. Moreover, the color varies according to the quantity of the test added; and if the latter be in excess, any of the varieties when viewed by transmitted light, will seem of a red color. If, however, the solution be subsequently plentifully diluted with water, the green tint is rendered evident.

Thomson remarks, first, that a solution of isinglass gives a more abundant precipitate in the Indian than in the Russian rhubarb; and second, that the decoction of yellow bark gives a more abundant *greenish* precipitate with the infusion of Russian than with the East Indian rhubarb, in the latter of which the precipitate is bright yellow.

These tests do not appear to me to be conclusive. The precipitate caused by the solution of isinglass, gives a bulkier but not really more abundant precipitate in the one case than in the other, as is evident when the precipitate has subsided, and the difference of color in the other test is so small that it requires a somewhat artistic eye to appreciate the distinction, and it certainly would not serve otherwise than as a comparative test. I may incidentally mention here, that in the course of my experiment, I found that the acetate of lead, when ammonia had previously been added, gave with infusion of Indian rhubarb a violet-red precipitate, and one approaching to a brick-red with Russian rhubarb, the precipitate in the latter being of a coarser description than in the former. The same objection, however, applies to this as to the previously cited tests, for any reagent depending for its efficiency on a slight variation in color or bulk, must practically be of little value. Bearing this in mind, I have endeavored to find one not liable to these objections.

The test to which I am about to direct your attention is based on the supposition which I had the honor of suggesting to you some two years since, that the precipitate formed in the tincture of rhubarb is the result of the oxidation of the active principle of the rhubarb. I am not at present prepared to maintain the correctness or inaccuracy of this view, but I am now engaged in some experiments on the subject, which, if satisfactory, I shall have the pleasure of submitting to you on a future day. Starting then with this hypothesis, and remarking that the tincture, when made with Indian, deposited more than with the Russian rhubarb, I thought it probable that an oxidizing agent might exhibit sufficient difference in its *modus agendi* on the species of rhubarb to enable us to identify them thereby. In this, though at first unsuccessful, my anticipations were ultimately realized. If two drachms of simple (*proof*) tincture, of the P. L. strength, be placed in a test tube, and treated with one drachm of a mixture of equal parts of nitric acid and distilled water, the following results will take place:—

East Indian soon becomes cloudy, and in from five to twenty minutes is turbid.

Russian remains unchanged for three or four hours.

English loses its brightness in half an hour; on holding it before a light a precipitate may be seen diffused through it.

If, instead of ʒij. of tincture, one of tincture and one of water be used, the test as regards the Indian is still more prompt, but the difference between the English and Turkey not so clearly defined.

There are one or two precautions to be observed in the application of this test: the dilute acid should be gradually added, the test tube being shaken in the meantime. Again, if rectified tincture or strong acid be used, very equivocal results are obtained. My first experiments were made with strong acid and rectified tincture. The results were excellent, as far as regarded three or four samples; but when I came to essay another, the very reverse of the former experiments took place. I thought at first that the samples of rhubarb could not be rightly marked, but on again returning to the former samples, they also gave contradictory results. I procured other samples, but ultimately discovered that this anomalous reaction was due to the time the nitric acid was left in contact with the supertangent film of tincture, in which it seemed to set up a sort of fermentation, which, after the two were mixed by agitation, propagated itself throughout the liquid. Thus, if two drachms of tincture of East Indian rhubarb be placed in two separate test tubes, held in the hand, and the acid added; if the tube to which the acid is last added be immediately agitated, and then the other treated in a similar manner, the one will shortly let fall a ropy deposit, while the other remains clear. By using the dilute acid and proof tincture these fallacies are avoided.—*Lond. Pharm. Journ.*, Feb. 1853.

